# Supplementary material for: Molecular Transmission Dynamics of Primary HIV Infections in Lazio Region, Years 2013–2020
Source: Viruses. 2021 Jan 25;13(2):176. doi: 10.3390/v13020176 (PMC7911907; doi:10.3390/v13020176)
Supplement: Supplementary file 1 [file viruses-13-00176-s001.pdf]

Supplementary Table S1:

Accession number for the 17 B reference sequences:

B.FR.83.HXB2_LAI_IIIB_BRU.K03455

B.NL.86.3202A21_ACH3202A21.U34604

B.GA.88.OYI_397.M26727

B.EC.89.EC003.AY173959

B.KR.92.HP_10_02SHJ8_6986.KJ140255

B.TW.94.TWCYS_LM49.AF086817

B.GE.98.98GEMZ003.DQ207943

B.CU.99.Cu19.AY586542

B.AR.00.ARMS008.AY037269

B.CN.01.CNHN24.AY180905

B.BR.02.02BR002.DQ358805

B.AU.03.PS2008_Day182.DQ676875

B.CY.05.CY018.FJ388890

B.CA.06.502_1027_wg01.JF320413

B.PE.07.502_2649_wg8.JF320019

B.PL.08.BP00074_LH01.JN687691

B.RU.11.11RU21n.JX500708
